# Supplementary material for: Specificity in glycosylation of multiple flagellins by the modular and cell cycle regulated glycosyltransferase FlmG
Source: eLife. 2020 Oct 27;9:e60488. doi: 10.7554/eLife.60488 (PMC7591256; doi:10.7554/eLife.60488)
Supplement: Supplementary file 1. — LacZ activity of BACTH assay. [file elife-60488-supp1.docx]

**Table S1. LacZ activity of BACTH assay.**

|  |  | **LacZ activity (Miller Unit)** | |
| --- | --- | --- | --- |
|  |  |  |  |
| **Combination in BTH101 strain** |  | **Mean** | **Standard deviation** |
|  |  |  |  |
| pUT18C + pKNT25 |  | 127 | 35 |
|  |  |  |  |
| pUT18 + pKNT25 |  | 117 | 25 |
|  |  |  |  |
| pUT18 + pKT25 |  | 149 | 74 |
|  |  |  |  |
| pUT18C + pKT25 |  | 118 | 49 |
|  |  |  |  |
|  |  |  |  |
| pKNT25-FlmG + pUT18C-FljJ |  | 3277 | 88 |
|  |  |  |  |
| pKNT25-TPR + pUT18C-FljJ |  | 2723 | 196 |
|  |  |  |  |
| pKNT25-GT + pUT18C-FljJ |  | 63 | 22 |
|  |  |  |  |
| pKT25-FlmG + pUT18C-FljJ |  | 5219 | 351 |
|  |  |  |  |
| pKT25-TPR + pUT18C-FljJ |  | 4178 | 367 |
|  |  |  |  |
| pKT25-GT + pUT18C-FljJ |  | 53 | 7 |
|  |  |  |  |
|  |  |  |  |
| pKNT25-FlmG + pUT18C-FljK |  | 2891 | 755 |
|  |  |  |  |
| pKNT25-TPR + pUT18C-FljK |  | 2815 | 315 |
|  |  |  |  |
| pKNT25-GT + pUT18C-FljK |  | 79 | 13 |
|  |  |  |  |
| pKT25-FlmG + pUT18C-FljK |  | 8062 | 3526 |
|  |  |  |  |
| pKT25-TPR + pUT18C-FljK |  | 4130 | 459 |
|  |  |  |  |
| pKT25-GT + pUT18C-FljK |  | 78 | 42 |
|  |  |  |  |
|  |  |  |  |
| pKNT25-FlmG + pUT18C-FljM |  | 3936 | 1020 |
|  |  |  |  |
| pKNT25-TPR + pUT18C-FljM |  | 3321 | 163 |
|  |  |  |  |
| pKNT25-GT + pUT18C-FljM |  | 87 | 6 |
|  |  |  |  |
| pKT25-FlmG + pUT18C-FljM |  | 7961 | 740 |
|  |  |  |  |
| pKT25-TPR + pUT18C-FljM |  | 6264 | 551 |
|  |  |  |  |
| pKT25-GT + pUT18C-FljM |  | 85 | 14 |
|  |  |  |  |
