# Supplementary material for: Specificity in glycosylation of multiple flagellins by the modular and cell cycle regulated glycosyltransferase FlmG
Source: eLife. 2020 Oct 27;9:e60488. doi: 10.7554/eLife.60488 (PMC7591256; doi:10.7554/eLife.60488)
Supplement: Supplementary file 2. — Strains and plasmids used in this study. [file elife-60488-supp2.docx]

**Table S2. Strains and plasmids used in this study**

| ***Caulobacter crescentus*** | **Relevant characteristics** | **Reference or source** |
| --- | --- | --- |
| NA1000 | syn-1000, synchronizable variant of strain CB15 | (Evinger 1977) |
| *neuB::Tn* (NS7) | NA1000 derivative with *himar1* insertion in *neuB*, Kana^R^ | This work |
| Δ*neuB* | NA1000 with in-frame deletion of *neuB* | This work |
| Δ*flmG* | NA1000 with in-frame deletion of *flmG* | This work |
| Δ*flj*^x6^ | NA1000 derivative with in-frame deletion of *fljJ*, *fljK*, *fljL* and *fljMNO* | (Faulds-Pain 2011) |
| Δ*neuB* Δ*flj*^x6^ | NA1000 derivative with in-frame deletion of *fljJ*, *fljK*, *fljL*, *fljMNO* and *neuB* | This work |
| *flmA::Tn* (NS148) | NA1000 derivative with *EzTn5* insertion in *flmA*, Kana^R^ | This work |
| *flmB::Tn* (NS76) | NA1000 derivative with *himar1* insertion in *flmB*, Kana^R^ | This work |
| Δ*flmH* | NA1000 with in-frame deletion of *flmH* | This work |
| Δ*CCNA_01531* | NA1000 with in-frame deletion of *CCNA_01531* | This work |
| Δ*CCNA_01537* | NA1000 with in-frame deletion of *CCNA_01537* | This work |
| Δ*flmH* Δ*1531* Δ*1537* | NA1000 with in-frame deletion of *flmH*, *CCNA_01531* and *CCNA_01537* | This work |
| Δ*flmD* | NA1000 with in-frame deletion of *flmD* | This work |
| NA1000 *ctrA401* | NA1000 derivative with the temperature-sensitive *ctrA401* allele | (Quon 1996) |
| Δ*mucR1* Δ*mucR2* | NA1000 derivative with in-frame deletion of *mucR1* and *mucR2* | (Fumeaux 2014) |
| UG1277 | Δ*mucR1* Δ*mucR2* carrying the *sciP* T24I allele | (Fumeaux 2014) |
| UG1278 | Δ*mucR1* Δ*mucR2* carrying the *sciP* T65A allele | (Fumeaux 2014) |
| UG1280 | Δ*mucR1* Δ*mucR2* carrying the *ctrA* T170A allele | (Fumeaux 2014) |
| *flbD::Tn* (NS228) | NA1000 derivative with Hyper*Mu* insertion in *flbD*, Kana^R^ | This work |
| *flaF::Tn* (NS273) | NA1000 derivative with Hyper*Mu* insertion in *flaF*, Kana^R^ | This work |
| *flaF::Tn* (NS307) | NA1000 derivative with Hyper*Mu* insertion in *flaF*, Kana^R^ | This work |
| *rpoN::Tn* (NS229) | NA1000 derivative with Hyper*Mu* insertion in *rpoN*, Kana^R^ | This work |
| *rpoN::Tn* (NS272) | NA1000 derivative with Hyper*Mu* insertion in *rpoN*, Kana^R^ | This work |
| Δ*neuB rpoN::Tn* | NA1000 derivative with in-frame deletion of *neuB* and Hyper*Mu* insertion in *rpoN* (NS229), Kana^R^ | This work |
| Δ*neuB rpoN::Tn* | NA1000 derivative with in-frame deletion of *neuB* and Hyper*Mu* insertion in *rpoN* (NS272), Kana^R^ | This work |
| ***Sinorhizobium fredii*** |  |  |
| NGR234 | Wild-type strain | (Stanley 1988) |
| NGR234Δ*rkpQ* | NGR234 with in-frame deletion of *rkpQ* | This work |
| NGR234Δ*rkp3_013* | NGR234 with in-frame deletion of *rkp3_013* | This work |
| ***Escherichia coli*** |  |  |
| EC100D | Cloning strain | Epicentre Technologies |
| S17-1 | For plasmid mobilization | (Simon 1983) |
| Rosetta™(DE3)pLysS | BL21 derivative for protein expression from T7 promoter | Novagen |
| BTH101 | F^−^ *cya-99 araD139 galE15 galK16 rpsL1* (Str^r^) *hsdR2 mcrA1 mcrB1* | Euromedex |
| **Plasmid** | **Relevant characteristics** | **Reference or source** |
| pNPTS138 | Suicide vector used for gene replacement; Kana^R^ | M.R.K. Alley |
| pNPTS_Δ*neuB* | pNPTS138 derivative carrying the in-frame deletion of *neuB*; Kana^R^ | This work |
| pSA37 | pNPTS138 derivative carrying the in-frame deletion of *flmG*; Kana^R^ | This work |
| pSA252 | pNPTS138 derivative carrying the in-frame deletion of *flmH*; Kana^R^ | This work |
| pSA265 | pNPTS138 derivative carrying the in-frame deletion of *CCNA_01531*; Kana^R^ | This work |
| pSA617 | pNPTS138 derivative carrying the in-frame deletion of *CCNA_01537*; Kana^R^ | This work |
| pSA253 | pNPTS138 derivative carrying the in-frame deletion of *flmD*; Kana^R^ | This work |
| pSA35 | pNPTS138 derivative carrying the in-frame deletion of *rkpQ*; Kana^R^ | This work |
| pK18*mobsacB* | Suicide vector used for gene replacement; Kana^R^ | (Schafer 1994) |
| pSA326 | pK18*mobsacB* derivative carrying the in-frame deletion of *rkp3_013*; Kana^R^ | This work |
| pOK12 | Cloning vector; Kana^R^ | (Vieira 1991) |
| pSA58 | pOK12 derivative carrying the *neuB* ORF; Kana^R^ | This work |
| pSA90 | pOK12 derivative carrying the *neuB* E30A variant; Kana^R^ | This work |
| pSA91 | pOK12 derivative carrying the *neuB* H245A variant; Kana^R^ | This work |
| pSA92 | pOK12 derivative carrying the *neuB* R322A variant; Kana^R^ | This work |
| pMT335 | Medium copy number plasmid for inducible expression; P*_van_*, Gm^R^ | (Thanbichler 2007) |
| pSA53 | pMT335 derivative carrying the *neuB* ORF; Gm^R^ | This work |
| pSA93 | pMT335 derivative carrying the *neuB* E30A variant; Gm^R^ | This work |
| pSA94 | pMT335 derivative carrying the *neuB* H245A variant; Gm^R^ | This work |
| pSA95 | pMT335 derivative carrying the *neuB* R322A variant; Gm^R^ | This work |
| pSA59 | pMT335 derivative carrying the *flmG* ORF (full-length); Gm^R^ | This work |
| pSA645 | pMT335 derivative carrying the *flmG* GT domain (residues 309-596); Gm^R^ | This work |
| pSA126 | pMT335 derivative carrying the *neuB1^Cj^* ORF; Gm^R^ | This work |
| pSA47 | pMT335 derivative carrying the *neuB2^Cj^* ORF; Gm^R^ | This work |
| pSA48 | pMT335 derivative carrying the *neuB3^Cj^* ORF; Gm^R^ | This work |
| pSA42 | pMT335 derivative carrying the *S. fredii* *rkpQ* ORF; Gm^R^ | This work |
| pSA263 | pMT335 derivative carrying the *S. fredii rkpO* ORF; Gm^R^ | This work |
| pSA569 | pMT335 derivative carrying the *S. fredii rkpL* ORF; Gm^R^ | This work |
| pSA568 | pMT335 derivative carrying the *S. fredii rkpM* ORF; Gm^R^ | This work |
| pSA570 | pMT335 derivative carrying the *S. fredii rkpLM* ORFs; Gm^R^ | This work |
| pSA60 | pMT335 derivative carrying the *fljK* ORF; Gm^R^ | This work |
| pSA107 | pMT335 derivative carrying the *fljK* ORF (codon optimised for *E. coli*); Gm^R^ | This work |
| pSA235 | pMT335 derivative carrying the *fljK* (codon optimised for *E. coli*) and *flmG* ORFs; Gm^R^ | This work |
| pSA283 | pMT335 derivative carrying the *S. fredii rkp3_013* ORF; Gm^R^ | This work |
| pSA290 | pMT335 derivative carrying the *flmH* ORF; Gm^R^ | This work |
| pSA292 | pMT335 derivative carrying the *CCNA_01531* ORF; Gm^R^ | This work |
| pSRK-Gm | pBBR1MCS-5 derived broad host range vector containing *lac* promoter, *lacI*^q^, l*acZ*α^+^; Gm^R^ | (Khan 2008) |
| pSA454 | pSRK-Gm derivative carrying the *fljK* ORF (codon optimised for *E. coli*); Gm^R^ | This work |
| pSA496 | pSRK-Gm derivative carrying the *C. crescentus* *flmG* ORF; Gm^R^ | This work |
| pSA236 | pSRK-Gm derivative carrying the *fljK* ORF (codon optimised for *E. coli*) and the *C. crescentus flmG* ORF; Gm^R^ | This work |
| pSRK-Km | pBBR1MCS-2 derived broad host range vector containing *lac* promoter, *lacI*^q^, l*acZ*α^+^; Kana^R^ | (Khan 2008) |
| pSA571 | pSRK-Km derivative carrying the *C. crescentus* *flmH* ORF; Kana^R^ | This work |
| pSA572 | pSRK-Km derivative carrying the *C. crescentus* *CCNA_01531* ORF; Kana^R^ | This work |
| pSA624 | pSRK-Km derivative carrying the *C. crescentus* *CCNA_01537* ORF; Kana^R^ | This work |
| pSA573 | pSRK-Km derivative carrying the *S. fredii* *rkp3_013* ORF; Kana^R^ | This work |
| pMT463 | Medium copy number plasmid for inducible expression; P*_xyl_*, Gm^R^ | (Thanbichler 2007) |
| pSA104 | pMT463 derivative carrying the *fljK* ORF; Gm^R^ | This work |
| pMT464 | Medium copy number plasmid for inducible expression; P*_xyl_*, Kana^R^ | (Thanbichler 2007) |
| pMT464_*fljJ* | pMT464 derivative carrying the *C. crescentus fljJ* ORF; Kana^R^ | (Faulds-Pain 2011) |
| pMT464_*fljK* | pMT464 derivative carrying the *C. crescentus fljK* ORF; Kana^R^ | (Faulds-Pain 2011) |
| pMT464_*fljL* | pMT464 derivative carrying the *C. crescentus fljL* ORF; Kana^R^ | (Faulds-Pain 2011) |
| pMT464_*fljM* | pMT464 derivative carrying the *C. crescentus fljM* ORF; Kana^R^ | (Faulds-Pain 2011) |
| pMT464_*fljN* | pMT464 derivative carrying the *C. crescentus fljN* ORF; Kana^R^ | (Faulds-Pain 2011) |
| pMT464_*fljO* | pMT464 derivative carrying the *C. crescentus fljO* ORF; Kana^R^ | (Faulds-Pain 2011) |
| pUCIDT-*flm* | pIDT carrying the synthetic sequence encoding *C. crescentus flmA*, *flmB*, *flmH*, *flmD*, *neuB* and *flmC* (codon-optimized for *E. coli*); Amp^R^ | This work |
| pET28a | T7 expression plasmid; Kana^R^ | Novagen |
| pSA44 | pET28a derivative expressing His_6_-NeuB; Kana^R^ | This work |
| pET47b | T7 expression plasmid; Kana^R^ | Novagen |
| pSA106 | pET47b derivative expressing *C. crescentus* FljK; Kana^R^ | This work |
| pCWR547 | pET-28a derivative expressing His_6_-SUMO-KidO | (Radhakrishnan 2010) |
| pSA363 | pCWR547 derivative expressing His_6_-SUMO-FlmG_(301-500)_; Kana^R^ | This work |
| pNKT25 | Low copy plasmid with T25 adenylate cyclase fragment Nter, Kan^R^ | Euromedex |
| pKT25 | Low copy plasmid with T25 adenylate cyclase fragment Cter, Kan^R^ | Euromedex |
| pUT18 | Low copy plasmid with T18 adenylate cyclase fragment Nter, Amp^R^ | Euromedex |
| pUT18C | Low copy plasmid with T18 adenylate cyclase fragment Cter, Amp^R^ | Euromedex |
| pNK92 | pKNT25-*flmG-GT*, Kan^R^ | This work |
| pNK93 | pKT25-*flmG-GT*, Kan^R^ | This work |
| pNK95 | pKNT25-*flmG*, Kan^R^ | This work |
| pNK96 | pKT25-*flmG*, Kan^R^ | This work |
| pNK98 | pKNT25-*flmG-TPR*, Kan^R^ | This work |
| pNK99 | pKT25-*flmG-TPR*, Kan^R^ | This work |
| pNK16 | pUT18C-*fljJ*, Amp^R^ | This work |
| pNK144 | pUT18C-*fljK*, Amp^R^ | This work |
| pNK330 | pUT18-*fljM*, Amp^R^ | This work |
| pRKlac290 | *lacZ* transcriptional fusion vector, pRK290 derivative; Tet^R^ | (Gober 1992) |
| pRKlac290_P*_neuB_* | pRKlac290 derivative carrying P*_neuB_-lacZ*; Tet^R^ | This work |
| pRKlac290_P*_flmG_* | pRKlac290 derivative carrying P*_flmG_-lacZ*; Tet^R^ | This work |
| pRKlac290_P*_flmA_* | pRKlac290 derivative carrying P*_flmA_-lacZ*; Tet^R^ | This work |
