# Supplementary material for: Specificity in glycosylation of multiple flagellins by the modular and cell cycle regulated glycosyltransferase FlmG
Source: eLife. 2020 Oct 27;9:e60488. doi: 10.7554/eLife.60488 (PMC7591256; doi:10.7554/eLife.60488)
Supplement: Supplementary file 3. — Oligonucleotides and synthetic genes used in this study. [file elife-60488-supp3.docx]

**Supplementary Table S3. Oligonucleotides and synthetic genes used in this study**

| **Primer** | **Sequence 5’-3’ (Restriction sites underlined)** |
| --- | --- |
| neuB_ko1 | AAA AAA GAA TTC ATC CAT TGG GCG ATC GCG TCC TCA A |
| neuB_ko2 | AAA AAA GGA TCC CTT GCG GCC GGC GAT CTC GAT AGA A |
| neuB_ko3 | AAA AAA GGA TCC GGC GAG CCG CTG GAC TGG TCG AT |
| neuB_ko4 | AAA AAA AAG CTT GCC TGC CGG TGA CCT GGG CGG CTT A |
| neuB_out1 | GCG GAA TCC CGA ATT GAA CGG AA |
| neuB_out2 | TGC CCG ACC CGA CGG CGG CGG GGA A |
| flmG_ko1 | AAA AGC TTC CGG CTC AAC ATC AG |
| flmG_ko2 | AAG GAT CCT GCC GAG GAT TCC AGG GCG C |
| flmG_ko3 | AAG GAT CCG CTC TGC GCG ATT TCG CC |
| flmG_ko4 | AAG AAT TCG TTG CCG GAA CAG CGC CTC |
| flmG_out5 | GGC TCA GCG AAA TGA TGC TGG |
| flmG_out3 | AAG TGC CTT CGA AGC GCT GG |
| flmH_ko1 | AAA GAA TTC CGG GCC GCG CCC TAT ATC GAG AA |
| flmH_ko2 | AAA AAA TCT AGA ATT CCG CAG TCT CAC CGC CAC CA |
| flmH_ko3 | AAA AAA TCT AGA TAC ACG ATC CCC GAT CTC ACC T |
| flmH_ko4 | AAA AAA AAG CTT CCT GCA TCG TCT CGG CTT CGG GGA A |
| flmH_out5 | GGC GGG CTC GAT CGT GTT CTT |
| flmH_out3 | CAG GCG CTC GAT GGC ACG GAT |
| 1531_ko1 | AAA GAA TTC GGT GAA GCC ATC TCG ACG GCC AA |
| 1531_ko2 | AAA GGA TCC GTC CTC GTC CAA CAA GTC TCG CA |
| 1531_ko3 | AAA GGA TCC CGC GCA CTG TCG GAC GCG CAC CTT |
| 1531_ko4 | AAA AAG CTT CGC TGA ACC GAG AAG TCG GTG TT |
| 1531_out1 | GCT AGC CGC CGA GCC TGA GAA |
| 1531_out2 | CCA GCG AAC GCG CGT AGA GAA |
| 1537_ko1 | AAA GCT AGC GAA GCG GCG ATA GCT GTC ATA |
| 1537_ko2 | AAA GGA TCC CGA GGT TCC GAG TCG GAT GAT |
| 1537_ko3 | AAA GGA TCC AAG CCT AAG GAG CTG CTG CCT T |
| 1537_ko4 | AAA AAG CTT GAC CTA TGC CAA GCG CGC TT |
| 1537_out1 | GCG TCC TGC GGG AAC GGC TT |
| 1537_out2 | CGA CAG GCC GAT ACG CTC ATA |
| flmD_ko1 | AAA AAA GAA TTC TGC GAC CGG CTT AAA AAG CGC ACA AA |
| flmD_ko2 | AAA AAA GGA TCC CGC GCA GAC GAA GAC GAT GCG CAA |
| flmD_ko3 | AAA AAA GGA TCC CGG GTG GCG GAG GCG TTT TTG GGT GT |
| flmD_ko4 | AAA AAA AAG CTT GCT GGT GCA GGC CAC GCT TGA AGA A |
| flmD_out5 | TCG GCG GCC CGA TTG ACC AA |
| flmD_out3 | GTC GCT GTT GGG CGA GAC GAA |
| rkpQ_ko1 | AA GAA TTC CGT GCT GTC CAA CGT TGA CG |
| rkpQ_ko2 | AA GGA TCC AGC GAT GGT CAA AAC TCT G |
| rkpQ_ko3 | AA GGA TCC CCG GTT ACA AAG GAT TCG GTC |
| rkpQ_ko4 | AA AAG CTT GGC AAA GCT AGC CGT GCA GC |
| rkpQ_out5 | CCT GCA TGA ATT CGA TGC CGC |
| rkpQ_out3 | CTC GCT TCG CTC TAC ATG GC |
| 013_ko1 | AAA GAA TTC GGC GGG GCT GGC TCG CGA TT |
| 013_ko2 | AAA TCT AGA GTC CAT TGT CGC TTT GCG AAG TT |
| 013_ko3 | AAA TCT AGA GAT TAA GAA GCG ATC TCG TCG TT |
| 013_ko4 | AAA AAG CTT GTT GCC GTG TCG CGC AGA GTA |
| 013_out5 | GTT TCA CAT TTC GGT GAT CGA CTT |
| 013_out3 | GAC ATG TAC ATC ATC GGA AAA CTT |
| neuB_E30A | GCC CTA TGT GAT CTG CGC GCT GTC GGG GAA CCA TAA C |
| neuB_E30A_as | G TTA TGG TTC CCC GAC AGC GCG CAG ATC ACA TAG GGC |
| neuB_H245A | CTG CGC GGT CGA AAA GGC TTT CAC CCT GGC CCG C |
| neuB_H245A_as | G CGG GCC AGG GTG AAA GCC TTT TCG ACC GCG CAG |
| neuB_R322A | CCA ATG TCC GCT CGG TCG CCC CCG GCA ACG GCT TGC |
| neuB_R322A_as | GCA AGC CGT TGC CGG GGG CGA CCG AGC GGA CAT TGG |
| neuB_N | AA CAT ATG TCC GCC CCC TCC ACC |
| neuB_E | AA GAA TTC CTA CCC CAC CAT CGA CCA G |
| FlmG_N | AA CAT ATG TCC CGT AAA AGC GCC C |
| FlmG_E | AA GAA TTC TCA GGA TGC GGC GAA ATC G |
| FlmG_int_N | AAA CAT ATG TCA GGA CAG GAC CTC AGG GGC AA |
| NeuB_Cj1_N | AA CAT ATG CAA ATA AAA ATA GAT AAA TTA ACT A |
| NeuB_Cj1_E | AA GAA TTC TCA TTC AAA ATC ATC CCA TGT TAG |
| NeuB_Cj2_N | AA CAT ATG AAA AAA ACT TTA ATC ATC GCA G |
| NeuB_Cj2_E | AA GAA TTC TTA CTC ACG GAT AAG CTC ATC T |
| NeuB_Cj3_N | AA CAT ATG CAA ATA GGA AAT TTT AAC ACC G |
| NeuB_Cj3_E | AA GAA TTC TCA TTG GAA ATC TCC TTG TTT TAA AG |
| RkpQ_N | AA CAT ATG AGC AGA GTT TTG ACC ATC GC |
| RkpQ_E | AA GAA TTC TCA AAA TTC GAC CGA ATC CT |
| RkpO_N | AAA AAA CAT ATG AAT TCG ATG CCG CCC GCT CAT AGA |
| RkpO_M | AAA AAA CAA TTG TTA GAC ACT ACG ACG GAG GCC GTC CGC AA |
| RkpL_N | AAA CAT ATG ATT TCT GGC AGC ACA ATA CTT |
| RkpL_E | AAA GAA TTC GCT TAA AGG CTC CCG ATC TTT |
| RkpM_N | AAA CAT ATG ATC CCC TAC GGG CGC CAA |
| RkpM_E | AAA GAA TTC ATG TCG TCA GTT CCG CCG T |
| FlmG_rbs_E | GAA TTC AGG AGG TAA AAA AAT GTC CCG TAA AAG CGC CCT |
| FlmG_X | AAA TCT AGA TTA GGA TGC GGC GAA ATC GCG CA |
| FlmH_N | AAA CAT ATG AGG ACC ATG GTG GCG GTG A |
| FlmH_E | AAA GAA TTC AGG CCG CGC GGC AGG TGA |
| 1531_N | AAA CAT ATG ATC GAG CTG CGA GAC TTG TT |
| 1531_E | AAA GAA TTC TTA AGC GGC GAT AAG GTG CGC GT |
| 1537_N | AAA CAT ATG GCG GCA CGG ATC ATC CGA |
| 1537_X | AAA TCT AGA CTA AGG CAG CAG CTC CTT AGG CTT |
| rkp3_013_N | AAA AAA CAT ATG GAA CTT CGC AAA GCG ACA A |
| rkp3_013_M | AAA AAA CAA TTG TTA ATC GAC GAA TTT GAC GA |
| FljK_N | AAA CAT ATG GCG CTG AAC AGC ATC AAT A |
| FljK_X | AAA TCT AGA TTA ACG GAA CAG GCT CAG GAT C |
| FlmG_547_N | AAA CAT ATG TTC CAG ATT CCT GGC GTC CGC T |
| FlmG_547_S | AAA GAG CTC ACA CGC CCA GCT CTT CCT TGG C |
| NK-74 | AAA AAA TCT AGA GAT GTC CCG TAA AAG CGC CCT |
| NK-75 | AAA AAA GGT ACC CCG GAT GCG GCG AAA TCG CG |
| NK-76 | AAA AAA GGT ACC CCG AGG TCC TGT CCT GAC CAG |
| NK-77 | AAA AAA TCT AGA GTC AGG ACA GGA CCT CAG GG |
| NK-78 | AAA AAA GGT ACC GAG GTC CTG TCC TGA CCA G |
| NK-79 | AAA AAA GGT ACC TCA GGA TGC GGC GAA ATC G |
| NK-10 | AAA AAA TCT AGA GAT GGC GCT GAA CAG CAT CAA TAC |
| NK-11 | AAA AAA GGT ACC TTA ACG GAA CAG GCT CAG GAT C |
| NK-01 | AAA AAA TCT AGA GAT GGC GCT TAG CGT CAA CA |
| NK-02 | AAA AAA GGT ACC TTA CCC GCC CTT GAA CAG AG |
| NK-62 | AAA AAA TCT AGA GAT GGC GCT GAA CAG CAT CAA |
| NK-64 | AAA AAA GGT ACC TTA GCG GAA CAG GCT CAG GA |
| PneuB_E | AAA AAA GAA TTC GGC TTG ATC GGG CGG CGC GCT GGA A |
| PneuB_X | AAA AAA TCT AGA CCA CAG CCC GCC GTG GAT CTT GAA |
| PflmG_E | AAA AAA GAA TTC TGC ACG ACA GCG CCG TTA AGA A |
| PflmG_X | AAA AAA TCT AGA AAT CGC CGG TCT TCT CGC GTG CAA |
| PflmA_E | AAA GAA TTC AGA CCA TCA AGG TCT GGG CGA A |
| PflmA_X | AAA TCT AGA CTG CTT CAA TTC GTC GCG GGA A |
| **Synthetic DNA** (from Integrated DNA Technologies, Coralville, Iowa, USA) | |
| *C. crescentus fljK*, codon optimized for *E. coli* (5’-3’) | CATATGGCTCTGAACTCTATTAACACCAACGCCGGCGCTATGATTGCACTGCAGAACCTGAATGGTACCAACTCCGAGCTGACGACTGTTCAGCAGCGTATCAACACCGGTAAGAAAATTGCGTCCGCCAAAGATAACGGCGCAATCTGGGCTACCGCAAAAAACCAATCTGCCACTGCAGCTAGCATGAACGCTGTTAAAGACTCCCTGCAGCGTGGCCAGTCTACCATCGACGTTGCCCTGGCCGCCGGTGACACTATCACCGATCTGCTGGGTAAAATGAAAGAAAAGGCTCTGGCCGCGTCTGACACCTCCCTGAATACCGCGTCTTTTAACGCCCTGAAATCTGACTTCGATAGCCTGCGTGACCAGATCGAGAAGGCAGCGACTAATGCTAAATTCAACGGTGTTAGCATCGCCGACGGCTCTACCACTAAACTGACCTTCCTGGCCAACTCTGACGGTTCTGGTTTCACCGTTAACGCCAAAACTATTTCTCTGGCTGGTATCGGCCTGACCACTACCTCCACCTTTACCACCGCGGCGGCGGCGAAGACCATGATCGGCACTATCGATACTGCGCTGCAGACTGCCACCAACAAACTGGCCTCTCTGGGTACCTCCTCTGTGGGTCTGGACACGCATCTGACTTTTGTGGGCAAACTGCAGGATAGCCTGGATGCGGGCGTTGGTAACCTGGTGGATGCTGACCTGGCAAAGGAGTCTGCTAAGCTGCAATCTCTGCAGACTAAACAACAGCTGGGCGTTCAGGCACTGTCCATCGCCAACCAATCTTCTTCCTCTATTCTGTCTCTGTTTCGCTGAATTCTAGA |
| *C. crescentus flmA*, *flmB*, *flmH*, *flmD*, *neuB* and *flmC*, codon optimized for *E. coli* (5’-3’) | AGGATCCGGATGTGAGCGGATAACAATTACGAGCTTCATGCACAGTGAAATCATGAAAAATTTATTGGCTTTGTGAGCGGATAACAATTATAATATGTGGAAAGAAGGAGATACCATATGGGGCGTTTTAGCCCAAAGAGTTTGGATCTGGACGGAAAGGTTATCTTGGTAACGGGCGGTACTGGAAGCTTCGGGCGTCGTTTCATCGAGACTGTCTTGCGCCGTTACGATCCCCGCAAAGTTATCGTCTATTCGCGCGATGAATTAAAACAGAGTGACATGCAAATTGAGCTTCGCGAGCAATTCGATGAGGCCACCGTAGCAAAGATGCGTTTTTTTCTGGGCGACGTGCGTGATCGTGAGCGTTTAACGTTAGCGCTTCGTGGAGTCGACATTGTCATTCATGCAGCCGCACTTAAACAGGTACCAGCGGCAGAATATAATCCCTCCGAATGTATCCACACGAATGTGTTGGGTGCGGAAAACGTAGTATGGGCGTCACTGGCTAACGCCGTTAAGCAGGTGGTCGCCTTATCTACGGACAAAGCTTGTAATCCGACTAACCTGTATGGTGCAACGAAGTTGGCCTCTGACAAGACGTTCGTGGCTGCCAACAATCTGAGTGGAGACATCGGGACCCGCTTTTGCGTGGTTCGCTATGGTAACGTAGTCGGGTCTCGCGGCTCAGTAGTACCACTTTATCGTCGTCTGTTGAGCCAAGGGGCGACGGAGTTGCCAGTCACGGACCCTCGCATGACCCGCTTCTGGATTACGTTGAATGAGGGCGTGGACTTCGTACTTTCTTCATTGACCATGATGCGCGGAGGCGAGATTTTTGTGCCGAAGATCCCCAGTATGGCAATGCCTGATTTGGTAAAAGCCATGTCTAGCACTGCTGCAATGAAGGTAATCGGTATCCGCCCAGGAGAGAAACTTCATGAAATCATGATCAGCGCGGATGATGCCCGCAGCACCGTGGAGTTCGATGACCGCTATGCAATCGAACCGAATTTCGCAGAATTTGGCCGTGAGCCCTACGCAGCAAGTGACGGCGCTAAACCCGTGGCCGAGGACTTCAGCTACAGCTCAGACAATAATCATGACTGGTTGTCTCCCGAAGGCTTGTTAGCCATGTTAGAAGAGAAGGCCACGTGAAGATCTCCCCCGGGAAGAAGGAGATATACCATGACAGGCGGATTTTTACCTTATGGGCGTCAGACTATTGAGGAGGATGACATCGCTGCGGTAGCGGAAGCATTGCGCGGCGACTTTCTGACGACTGGCCCTACAGTGGAAGCTTTCGAGACAGCGTTCGCCGCTAAAGTCGGCGCTGATCACGCAATCGCGGTATCGAACGGAACAGCTACCTTGCACCTTGCCATGATGGCTCTTGGTATTGGTGAAGGTGATGTATGCGTAGCACCAAGTGTGACTTTTCTGGCAACCGCTAACTGTGCACGTTATGTAGGTGCGGAAGTAGTGTTCGCCGACGTTGACCCGGACAGTGGTCTTATGACACCAGACACCCTGGCGCGCGCTTTGGCAGGTGCACGTGATAAGCGTGTTAAAGCTGTACTTCCAGTACATCTGCGTGGGGACGTATGTGATCTTCCCGCGTTGAAAGCAATGGCATCAGCGAGCGGCGCCGTGCTTGTGGAAGATGCCCCGCATGCCCTGGGTTCGATCGCTACCTTTGATGGCGTAGCGCATCCAGTCGGAGATGGTGCGTACAGTTCATTCGCAAGTTTCTCCTTTCACCCCGTAAAGACGCTGGCCACAGGGGAGGGAGGGATGTTGACCACCAACGACCCCGCACTGGCCGCAAAGGCGCGTTTGCTTCGCAGTCACGGGATGGTCCGCCAGCCGGGTGGAGATCCGTGGTGGTACGAGATGCCCGAACTGGGATTCAATTACCGCATTCCTGATGTTTTATGTGCCTTAGGTTTATCCCAACTGGCGAAACTTGACCGTTTTGTTGCACGCCGTCGTGACCTTACTGCCCTTTACGCTCGCTTATTGGCGGAGCGCGCTCCCCGTGCGCGTTTAGCCACCAGCCCGGACCACTCAGACGCTGCCTTACACCTGTTGACGGTTTTAATTGATTTCGAGGCCGAGGGTATTTCCCGCCGTACCGTAGTTGAATCCCTTAAAACTCAAGGAGTAGGAACGCAGGTGCACTACATCCCGGTGCACCGTCAGCCATATTATGCACAGCGCTACGGGGTCGCCGACTTGCCCGGAGCTGACGCGTGGTACGCCCGTTGCTTAACCTTGCCGCTGTATCCAGCTATGACTAATGGAGACGTTGAGCGCGTTGTCGGTGCTTTAGCCACTGTTTTAGGGTGAGCTAGCGGAGCTCAAGAAGGAGATATACCATGGTTGCGGTGCGCCTTCGTAACCTGGTCGAATCTGATCGCGAACGCCTTCTTATTTGGCGCAACAGTCCAGATGTTAGCGCATATATGTACTCAGATCATAAGATTGGTCACGAAGAACATGACCACTGGTTCGACGTCGCGCGTCATGACCCACGTCGTCGCTACTGGATTATCGAGGCTGACGGGGAGCCGGTCGGTCTTGCCAATCTTGCTGACATTGATTTGGTTCACCGTCGCTGTGCTTGGGCCTACTACTTGGCAAGCCCCAAAGTGCGTGGACTGGGTGTCGGCAGTTTTGTTGAGTTCCAAATTATCGAATACGTTTTCAATCAGTTGCACCTGAACAAATTGTGGTGCGAAGTCCTTATCAGTAATGAATCCGTATGGCGTCTGCATGAACTTTACGGCTTCCAGCGCGAGGCTTTATTTCGCCAGCATGTTATGAAACAGGGCCATGAAGTGGACGTAATTGGTTTAGGACTGCTTGCCAGTGACTGGGCCGCTCGCCGCGATGCCATGGCCGAACGCTTGTGTGCGAAAGGATATACAATCCCCGACTTGACCTGCCGCGCGGCCTGAGATATCGCGGCCGCAAGAAGGAGATATACCATGTCCTTACGCATCGTCTTTGTATGCGCAGCCGGCCCATCTGTGGGTGGTGGACATGTCATGCGTTCCTTGACACTTGCACGCGCGTTAGCGGCGCGCGGAGCGACATGTGCGTTTTTGGGAACACCCGAGGTAGCAGCAGTCTTAGACGCCTTCGGTCCTGATATGGCGCGTGCCGACACCGCCGAGCCCTTCGAAGCTGTAGTCTTTGACTCCTATGCACTTACCGCGGACGACCATCGCCGTATCGCGGCGGGACGTCCCGCGTTAGTAATCGACGATTTAGCCGACCGCCCTCTTGCAGCAGACCTGGTGCTTGATGCTGGACCGGCTCGCCGCGCCGAGGATTACGCAGGACTGGTGCCCGCACATGCACGTCTTCTGCTGGGTCCGAATCACGCACCGGTCCGTCCAGCTTTTGTTGCGTTACGCGAGGCAGCCTTAGCACGCCGTGCGCAGCAGGGACCGGTACGTCGCATTCTTGTATCTCTGGGCATGACGGACGTGGGGGGAATTACAGGACGTGTGGTCGCACTTCTTGCCCCAATCCTTGGGGAGGTCACTCTGGATCTTGTGGTGGGAGCGGGAGCCCCGAGCTTGCCTGCTCTGCGTGCATTAGCCGCTGAAGACCCTCGCCTTGTTCTTCATATTGACACGCAGGATATGCCACGCCTTGTTCTTGAAGCCGACTTGGCCATCGGCGCAGGAGGTTCCACGACGTGGGAGCGCTGTGTCCTTGCCTTGCCAGCTTTGACTCTTATCTTAGCCGATAACCAAATTGCCGCGGCACGTGCTCTTGAAGCAGCTGGCGTAACCCCTTGTTTGGACGTAACAGCCCCGGATTTTGACACGGCCTTTGCAGCTCTTGCGCAGAACCTGATTGCTGATCCGGATCGTCGTGCCGCACTTAGTGCTGCCTCAGCTACGGTCTGTGATGGACGTGGCGCGGAGCGCGTGGCTGAAGCATTCTTGGGAGTCACCACCACATGACAATTGCTGCAGAAGAAGGAGATATACCATGTCCGCTCCGAGTACCGAGATCCCCCCTTCGATTGAGATTGCTGGACGCAAGATCGGGGCCGATCACAGCCCCTACGTTATCTGTGAGTTGTCGGGCAATCATAATGGGTCCCTTGAACGTTGCTTAGCTATGGTAGACGCTGCCGCAGATACCGGATGCGACGCCATCAAAATTCAAACTTACACAGCCGACACAATCACCTTGGATGTAGATCGTCCGGAGTTCAAAATCCACGGGGGATTGTGGGATGGACGCACTCTGTATGAGCTTTATGAGGAAGCTCATACTCCCTTTGAGTGGCACGCGGCCATCTTCGAACGCGCTCGTCAGCGCGGTGTCACGATTTTTTCTTCTCCATTTGACGAGACTGCCGTCGACCTTTTAGATTCGCTGGGGGCGCCAGCTTTTAAAATTGCAAGCTTTGAAGCGGTAGACCTTCCGCTTATCAAATACGCGGCAGCCAAAGGGAAACCCTTAATTATTTCCACTGGAATGGCGAACCTTACGGAGATGCAAACCGCCCTTGATACAGCTTTGTCAGCAGGCGCTCCGGGAGTGTTACTTTTACACTGTGTTTCTTCATACCCTGCTACGTTCGCAGACGCGAACGTCCGCACCGTGCCGGATATGGCGGCACGCTTCGGATGCCCGATTGGCCTTTCCGATCACACGCCCGGTACAGCAGCTAGTGTCGCCGCTGTGAGCTTAGGGGCGTGTGCAGTAGAAAAACATTTCACGCTTGCCCGTGCCGATGGTGGTCCGGACGCCGCATTCTCTCTTGAACCTGCGGAGTTTAAGGCATTAGTTGATGACACAAAGAATGCTTGGGCTGCGTTGGGACGTGCACACTACGATGTGCTGGGGTCAGAGGCAACATCACTGTTATTCCGCCGTTCTCTGTACGTTACAGCCGACGTGAAGGCTGGCGAACCTTTAACGCGCGCGAATGTTCGTTCAGTGCGTCCCGGCAATGGGTTGCCACCTGCGGATTTGGATAAAGTTCTGGCGGGAAAGGCAACCCGCGATCTTGCGCGCGGCGAGCCTCTTGACTGGTCAATGGTCGGTTGAAACTAGTACTTAAGAAGAAGGAGATATACCATGATCTTGGCCATCCTGCAAGCCCGCATGTCATCCACGCGCCTTCCTGGCAAGGTACTGATGCCCCTGCAACGCCAGCCCATGATCGTTCGTCAGATCGAACGTGTTGCCCGCTCAAAACGCATTGATAAGTTAGTTGTGGCCACGTCGGACCGCCCAGAGGACGATGCAATCGAAGCAGCCGTTCGCCGTGAAGGTATTGCGGTGTTTCGCGGGTCATTAGACAATGTCCAGCAGCGTTTTATTGGGGCATTGGATGCCCACCCTGCCGACCATGTAGTACGTCTGACCGCCGATTGTCCCCTTGCCGACCCGACACTTATTGATGCCACAATCGATTTATGTCTTTCAAAGGGCGCGGACTACGTATCTAATACACCGGAGGGTCACGCCCATCCAAAAGGGACCGATGTAGAGGTAATGACCGCAGCGGCATTGCGTCGCGCGGCTGCTGAAGCCACGACCAAAGAAGCATTTGAACACGTTACTTGGGACCTTTGGAACCAACCTCAACGCTGGACGTGTGCATGGTTGCCGTGCTTCCCAGATCAAGGAGCGGTACGCTGGACTGTGGATCGTCCGGATGATTATGCTTTTGTCGCTGCTGTATACGATGCCCTGTACCCAGCAAATCGCGCCTTTACGTCGGATGACATCCGTGCGTTTGTCGCTGGTCGCCCCGACCTGCAAGATTATGGTGGTGATCGCCGTGCATGAGAATTCCTCGGCCTCTAGAACTCGAG |
